# Supplementary material for: Insights into the Value of Lyso-Gb1 as a Predictive Biomarker in Treatment-Naïve Patients with Gaucher Disease Type 1 in the LYSO-PROOF Study
Source: Diagnostics (Basel). 2023 Aug 30;13(17):2812. doi: 10.3390/diagnostics13172812 (PMC10487050; doi:10.3390/diagnostics13172812)
Supplement: Supplementary file 1 [file diagnostics-13-02812-s001.zip › diagnostics-2530284-supplementary.pdf]

Supplementary Information

Insights into the dynamics of lyso-Gb1 accumulation in untreated patients with Gaucher disease type 1 in LYSO-PROOF study

A comprehensive list of all detected *GBA1* variants in the LYSO-PROOF study is provided in Table S1.

Table S1. Individual variants/alleles

No.	cDNA	allele	n	frequency in %
1	c.1226A>G	p.(Asn409Ser)	175	54.7
2	c.1448T>C	p.(Leu483Pro)	21	6.6
3	c.1603C>T	p.(Arg535Cys)	10	3.1
4	c.1342G>C	p.(Asp448His)	8	2.5
5	c.259C>T	p.(Arg87Trp)	8	2.5
6	c.1171G>A	p.(Val391Met)	8	2.5
7	c.1184C>T	p.(Ser395Phe)	8	2.5
8	c.1060G>A	p.(Asp354Asn)	6	1.9
9	c.479T>C	p.(Val160Ala)	6	1.9
10	c.667T>C	p.(Trp223Arg)	5	1.6
11	c.1177C>G	p.(Leu393Val)	4	1.3
12	c.1297G>T	p.(Val433Leu)	4	1.3
13	c.352A>G	p.(Lys118Glu)	4	1.3
14	c.84dupG	p.(Leu29Alafs*18)	4	1.3
15	c.1193G>A	p.(Arg398Gln)	3	0.9
16	c.371T>C	p.(Met124Thr)	3	0.9
17	c.1111C>A	p.(Pro371Thr)	2	0.6
18	c.1304A>C	p.(Asn435Thr)	2	0.6
19	c.475C>T	p.(Arg159Trp)	2	0.6
20	c.1296G>T	p.(Trp432Cys)	2	0.6
21	c.1300C>T	p.(Arg434Cys)	2	0.6
22	c.1316G>C	p.(Ser439Thr)	2	0.6
23	c.1331A>T	p.(Asp444Val)	2	0.6
24	c.1224G>A	p.((=))	2	0.6
25	c.1574G>A	p.(Gly525Asp)	2	0.6
26	c.1604G>A	p.(Arg535His)	2	0.6
27	c.253G>A	p.(Gly85Arg)	2	0.6
28	c.252_257dup	p.(Gly85_Arg86dup)	2	0.6
29	c.43_66del24bp	p.(Leu15_Ala22del)	2	0.6
30	c.786C>G	p.(Phe266Leu)	2	0.6
31	c.847T>C	p.(Tyr283His)	2	0.6
32	c.470_482delinsA	p.(Ile157_Pro161delinsAsn)	1	0.3
33	c.485T>C	p.(Met162Thr)	1	0.3
34	c.496G>T	p.(Asp166Tyr)	1	0.3
35	c.721G>A	p.(Gly241Arg)	1	0.3
36	c.849C>A	p.(Tyr283*)	1	0.3
37	c.544C>T	p.(Gln182*)	1	0.3

38	c.971G>A	p.(Arg324His)	1	0.3
39	c.222_224del	p.(Thr75del)	1	0.3
40	c.254G>A	p.(Gly85Glu)	1	0.3
41	c.653G>A	p.(Trp218*)	1	0.3
42	c.990G>A	p.(Trp330*)	1	0.3
43	c.492C>G	p.(Ser164Arg)	1	0.3
44	c.680A>G	p.(Asn227Ser)	1	0.3
total			320	100

A comprehensive list of all *GBA1* genotypes identified in the LYSO-PROOF study is provided in Table S2 including references for classification.

Table S2. Genotypes (at cDNA and protein level)

No.	genotype	n	frequency in %	classification and reference
1	c.1226A>G p.(Asn409Ser) homozygous	66	41.2	1 [1,2]
2	c.1226A>G p.(Asn409Ser) / c.1448T>C p.(Leu483Pro)	13	8.1	1 [1,2]
3	c.1226A>G p.(Asn409Ser) / c.1342G>C p.(Asp448His)	6	3.8	1 [1,2]
4	c.1603C>T p.(Arg535Cys) homozygous	5	3.1	-
5	c.1171G>A p.(Val391Met) homozygous	4	2.5	-
6	c.1226A>G p.(Asn409Ser) / c.1297G>T p.(Val433Leu)	4	2.5	1 [1,2]
7	c.1226A>G p.(Asn409Ser) / c.667T>C p.(Trp223Arg)	4	2.5	1 [1,2]
8	c.1060G>A p.(Asp354Asn) homozygous	3	1.9	-
9	c.1184C>T p.(Ser395Phe) homozygous	3	1.9	-
10	c.1226A>G p.(Asn409Ser) / c.84dupG p.(Leu29Alafs*18)	3	1.9	1 [1,2]
11	c.1448T>C p.(Leu483Pro) / c.259C>T p.(Arg87Trp)	3	1.9	-
12	c.479T>C p.(Val160Ala) homozygous	3	1.9	-
13	c.1177C>G p.(Leu393Val) homozygous	2	1.3	1 ClinVar
14	c.1226A>G p.(Asn409Ser) / c.1304A>C p.(Asn435Thr)	2	1.3	1 [1,2]
15	c.1226A>G p.(Asn409Ser) / c.475C>T p.(Arg159Trp)	2	1.3	1 [1,2]
16	c.1300C>T p.(Arg434Cys) / c.1342G>C p.(Asp448His)	2	1.3	-
17	c.1448T>C p.(Leu483Pro) / c.1224G>A p.((=))	2	1.3	-
18	c.259C>T p.(Arg87Trp) homozygous	2	1.3	1 ClinVar
19	c.352A>G p.(Lys118Glu) homozygous	2	1.3	-
20	c.1111C>A p.(Pro371Thr) homozygous	1	0.3	-
21	c.1184C>T p.(Ser395Phe) / c.1226A>G p.(Asn409Ser)	1	0.3	1 [1,2]
22	c.1184C>T p.(Ser395Phe) / c.1448T>C p.(Leu483Pro)	1	0.3	-
23	c.1193G>A p.(Arg398Gln) homozygous	1	0.3	1 [3]
24	c.1193G>A p.(Arg398Gln) / c.1448T>C p.(Leu483Pro)	1	0.3	-
25	c.1226A>G p.(Asn409Ser) / c.371T>C p.(Met124Thr)	1	0.3	1 [1,2]
26	c.1226A>G p.(Asn409Ser) / c.470_482delinsA p.(Ile157_Pro161delinsAsn)	1	0.3	1 [1,2]
27	c.1226A>G p.(Asn409Ser) / c.485T>C p.(Met162Thr)	1	0.3	1 [1,2]
28	c.1226A>G p.(Asn409Ser) / c.496G>T p.(Asp166Tyr)	1	0.3	1 [1,2]

29	c.1226A>G p.(Asn409Ser) / c.721G>A p.(Gly241Arg)	1	0.3	1 [1,2]
30	c.1226A>G p.(Asn409Ser) / c.849C>A p.(Tyr283*)	1	0.3	1 [1,2]
31	c.1226A>G p.(Asn409Ser) / c.544C>T p.(Gln182*)	1	0.3	1 [1,2]
32	c.1226A>G p.(Asn409Ser) / c.971G>A p.(Arg324His)	1	0.3	1 [1,2]
33	c.1296G>T p.(Trp432Cys) homozygous	1	0.3	-
34	c.1316G>C p.(Ser439Thr) homozygous	1	0.3	-
35	c.1331A>T p.(Asp444Val) homozygous	1	0.3	-
36	c.1448T>C p.(Leu483Pro) / c.222_224del p.(Thr75del)	1	0.3	-
37	c.1574G>A p.(Gly525Asp) homozygous	1	0.3	-
38	c.1604G>A p.(Arg535His) / c.84dupG p.(Leu29Alafs*18)	1	0.3	-
39	c.1604G>A p.(Arg535His) / c.990G>A p.(Trp330*)	1	0.3	-
40	c.252_257dup p.(Gly85_Arg86dup) homozygous	1	0.3	-
41	c.253G>A p.(Gly85Arg) homozygous	1	0.3	-
42	c.254G>A p.(Gly85Glu) / c.492C>G p.(Ser164Arg)	1	0.3	-
43	c.259C>T p.(Arg87Trp) / c.653G>A p.(Trp218*)	1	0.3	-
44	c.371T>C p.(Met124Thr) homozygous	1	0.3	-
45	c.43_66del24bp p.(Leu15_Ala22del) homozygous	1	0.3	-
46	c.667T>C p.(Trp223Arg) / c.680A>G p.(Asn227Ser)	1	0.3	-
47	c.786C>G p.(Phe266Leu) homozygous	1	0.3	-
48	c.847T>C p.(Tyr283His) homozygous	1	0.3	-
total		160	100	

References:

1. Grabowski GA, Zimran A, Ida H. Gaucher disease types 1 and 3: Phenotypic characterization of large populations from the ICGG Gaucher Registry. *Am J Hematol* 2015;90 Suppl 1:S12-8. doi: 10.1002/ajh.24063 [published Online First: 2015/06/23]
2. Pastores GM, Hughes DA. Gaucher Disease. In: Adam MP, Mirzaa GM, Pagon RA, et al., eds. GeneReviews((R)). Seattle (WA)1993.
3. Mozafari H, Tghikhani M, Rahimi Z, et al. Analysis of glucocerebrosidase (GBA) gene mutations in Iranian patients with Gaucher disease. *Iran J Child Neurol* 2021;15(3):139-66. doi: 10.22037/ijcn.v15i4.23834 [published Online First: 2021/07/21]

A comprehensive list regarding the frequency of the indicated clinical symptoms for all, type 1, mild type 1, severe type 1, and not classified patients in the LYSO-PROOF study is provided in Table S3.

Table S3. Frequency of presence of indicated clinical symptoms

	**Condition present**				
**Clinical symptoms**	**All patients N = 160**	**Type 1 N = 114**	**Mild type 1 N = 66**	**Severe Type 1 N = 48**	**Not classified N = 46**
	(%) N present/N total	(%) N present/N total	(%) N present/N total	(%) N present/N total	(%) N present/N total
Splenomegaly	(75.0) 117/156	(68.8) 77/112	(54.5) 36/66	(62.1) 41/46	(90.9) 40/44
Thrombocytopenia	(74.4) 116/156	(74.1) 83/112	(68.2) 45/66	(82.6) 38/46	(75.0) 33/44
Hepatomegaly	(62.4) 98/157	(53.6) 60/112	(39.4) 26/66	(73.9) 34/46	(84.4) 38/45

Anemia	(56.3) 89/158	(53.1) 60/113	(39.4) 26/66	(72.3) 34/47	(64.4) 29/45
Gaucher cells in Bone Marrow	(75.8) 75/99	(71.4) 45/63	(59.4) 19/32	(83.9) 26/31	(83.3) 30/36
Erlenmeyer flask' deformity of femur	(28.7) 43/150	(32.4) 34/105	(21.5) 14/65	(50.0) 20/40	(20.0) 9/45
Bone pain	(24.7) 39/158	(29.2) 33/113	(25.8) 17/66	(34.0) 16/47	(13.3) 6/45
Pancytopenia	(20.5) 32/156	(18.8) 21/112	(16.7) 11/66	(21.7) 10/46	(25.0) 11/44
Dyspnea	(6.9) 11/158	(5.3) 6/113	(4.5) 3/66	(6.4) 3/47	(11.1) 5/45
Osteonecrosis	(10.8) 16/148	(15.2) 16/105	(9.2) 6/65	(25.0) 10/40	(0.0) 0/43
Bone crises	(8.4) 13/154	(9.1) 10/110	(4.6) 3/65	(15.6) 7/45	(6.8) 3/44
Osteolytic lesions	(6.1) 9/147	(7.7) 8/104	(7.8) 5/64	(7.5) 3/40	(2.3) 1/43
Avascular necrosis of femoral head	(6.5) 10/153	(8.3) 9/109	(3.1) 2/65	(15.9) 7/44	(2.3) 1/44
Pathologic fractures	(5.8) 9/156	(7.2) 8/111	(9.2) 6/65	(4.3) 2/46	(2.2) 1/45
Seizures	(1.9) 3/157	(0.0) 0/112	(0.0) 0/66	(0.0) 0/46	(6.7) 3/45
Vertebral compression	(2.7) 4/148	(3.8) 4/104	(1.6) 1/64	(7.5) 3/40	(0.0) 0/44
Interstitial lung disease	(0.7) 1/152	(0.0) 0/110	(0.0) 0/64	(0.0) 0/46	(2.4) 1/42
Monoclonal gammopathy	(1.3) 2/149	(1.9) 2/105	(1.5) 1/65	(2.4) 1/41	(0.0) 0/44
Pulmonary hypertension	0.7) 1/148	(1.0) 1/105	(1.5) 1/65	(0.0) 0/41	(0.0) 0/43
Corneal pathology	(1.4) 2/144	(1.0) 1/101	(0.0) 0/63	(2.6) 1/38	(2.3) 1/43
Multiple myeloma	(0.0) 0/157	(0.0) 0/113	(0.0) 0/66	(0.0) 0/47	(0.0) 0/44

The following collaborators contributed with the participants recruitment and data collection to the LYSO-PROOF study (Table S4). Only collaborators that are not listed as co-authors are listed in Table S4.

Table S4: LYSO-PROOF Study Group

PI and Institution			
1	**Prof. Dr. Dimitrios Zafeiriou**<br>Aristotle University of Thessaloniki, Ippokration General Hospital, Pediatrics Department<br>Konstantinoupoleos, 54642, Thessaloniki, Greece	**10**	**Dr. Hanna Rosenbaum**<br>Head of Hematology clinic & Hematology Day care unit<br>Head of Gaucher center<br>The center for consultant medicine Clalit services<br>Nazareth towers, Nazareth, Israel
2	**Prof. Dr. Nezha Mouane**<br>Paediatrics' Gastroenterology Nutrition Department, Academic Children Hospital Ibn Sina<br>Mohammed V University, Rabat- Morocco	11	**Prof. Dr. Fadia Rahal**<br>Rheumatologist & Assistant Professor in Academic Medicine<br>Department of Rheumatology. Ben Aknoun Hospital<br>Academic Department: Faculty of Medicine. Algiers 1 University. Algiers 16306, Algeria
3	**Dr. Radha Rama Devi**<br>Prasad Research Foundation, 3-38-93, Plot No.7, Rukmini Devi Colony Annexe, West Marredpally<br>Secunderabad -500026 Telangana, Hyderabad, India	12	**Dr. Pilar Giraldo**<br>Unidad de Investigación Traslacional, Hospital Universitario Miguel Servet, FETEEG

			Pta Baja Edificio General, Pº Isabel La Católica 1-3, 50009 Zaragosa, Spain
4	**Prof. Dr. Sheela Nampoothiri**<br>Amrita Institute of Medical Sciences, Pediatric Genetics Department<br>- AIMS Ponekkara PO, Cochin 682041, India	13	**Dr. Souhila Melzi**<br>CHU Bab El Oued, Pediatrics Department<br>Boulevard Said Touati, Bab El Oued, 16008 Algier, Algeria
5	**Dr. Meenakshi Bhat**<br>Centre for Human Genetics, Centre for Human Genetics<br>Bangalore 560100, India	14	**Prof. Dr. Abdelkrim Berrah**<br>**Dr. Karima Daghor Abbaci**<br>CHU Bab El Oued, Internal Medicine, Univeristy of Algiers 1<br>Boulevard Said Touati, Bab El Oued, 16008 Algier, Algeria
6	**Prof. Dr. Paskal Cullufi**<br>University Hospital Center Mother Teresa, Pediatrics Department<br>Rruga e Dibrës 372, Tirana, 10001, Albania	15	**Dr. Abdelmadjid Benmansour (†) *(**deceased**)**<br>CHU Oran Centre Hospitalier et Universitaire d'Oran, Pediatrics Department<br>35 rue de Mostaganem, 31000 Oran, Algeria
7	**Dr. Xavier Solanich, MD, PhD**<br>Internal Medicine Department, Hospital Universitari de Bellvitge<br>IDIBELL, Barcelona, Spain	16	**Dr. Gustavo Adolfo Giraldo Ospina**<br>**Dr. Pilar Guatibonza Moreno**<br>ACMGen, Asociacion Colombiana de Médicos Genetistas - Bogotá, Colombia
8	**Prof. Dr.Ekram Fateen**<br>Biochemical Genetics Department<br>National Research Centre, Cairo Egypt	17	**Prof. Dr. Sondes Hadiji Mseddi**<br>Hospital Hedi Chaker, Route El Ain<br>3000, Sfax, Tunisia
9	**Prof. Dr. Elena Lukina**<br>National Medical Research Center for Hematology, Orphan Diseases Department 4<br>Novy Zykovsky pr.125167, Moscow, Russia	18	**Prof. Dr. Maciej Machaczka**<br>Karolinska University, Hospital Huddinge<br>Hematology Center, M54, SE-141 86, Stockholm, Sweden
